# Models of liaison psychiatry in different countries and the role of liaison psychiatrists as promotors of public and community mental health

**DOI:** 10.1192/j.eurpsy.2024.90

**Published:** 2024-08-27

**Authors:** F. Novais, D. Telles-Correia

**Affiliations:** ^1^Faculdade de Medicina, Universidade de Lisboa, Lisboa, Portugal

## Abstract

**Abstract:**

Consultation-Liaison (CL) psychiatry is the branch of psychiatric practice developed to offer support to patients with concomitant non-psychiatric diseases. In Portugal, most hospitals follow a model delivered by teams with Psychiatrists and Psychologists that support the medical team in wards. They act by advising directly other specialties’ colleagues after the observation of the patient and/or discussion of the case.

Bigger units, such as Santa Maria Hospital, in Lisbon, have tried a model of proximity to the community medical centers participating in local medical meetings, training of family doctors, discussing clinical situations directly and even doing psychiatric consultations, in community centers. This approach intends to extend primary mental health interventions and promote treatment in the community.

**Disclosure of Interest:**

None Declared

